# Integrins as the pivotal regulators of cisplatin response in tumor cells

**DOI:** 10.1186/s12964-024-01648-0

**Published:** 2024-05-13

**Authors:** Arya Nasimi Shad, Meysam Moghbeli

**Affiliations:** 1 Student Research Committee, Faculty of Medicine, Mashhad University of Medical Sciences, Mashhad, Iran; 2 Department of Medical Genetics and Molecular Medicine, School of Medicine, Mashhad University of Medical Sciences, Mashhad, Iran

**Keywords:** Integrin, Extracellular matrix, Microenvironment, Cisplatin, Prognosis

## Abstract

Cisplatin (CDDP) is a widely used first-line chemotherapeutic drug in various cancers. However, CDDP resistance is frequently observed in cancer patients. Therefore, it is required to evaluate the molecular mechanisms associated with CDDP resistance to improve prognosis among cancer patients. Integrins are critical factors involved in tumor metastasis that regulate cell-matrix and cell-cell interactions. They modulate several cellular mechanisms including proliferation, invasion, angiogenesis, polarity, and chemo resistance. Modification of integrin expression levels can be associated with both tumor progression and inhibition. Integrins are also involved in drug resistance of various solid tumors through modulation of the tumor cell interactions with interstitial matrix and extracellular matrix (ECM). Therefore, in the present review we discussed the role of integrin protein family in regulation of CDDP response in tumor cells. It has been reported that integrins mainly promoted the CDDP resistance through interaction with PI3K/AKT, MAPK, and WNT signaling pathways. They also regulated the CDDP mediated apoptosis in tumor cells. This review paves the way to suggest the integrins as the reliable therapeutic targets to improve CDDP response in tumor cells.

## Background

Tumor cell metastasis involves a series of sequential stages including spreading from the initial tumor, circulating through the bloodstream, and distant metastasis [1]. Tumor cells employ a variety of molecules to modulate their interactions with microenvironment. Integrins are critical factors involved in tumor metastasis that regulate cell-matrix and cell-cell interactions [2]. Extracellular matrix (ECM) is involved in oncogenesis, microenvironment remodeling, chemo-resistance, and tumor invasion [3, 4]. The integrin protein family comprises α- and β-subunits, which constitute cellular surface transmembrane receptors that are involved in cellular adhesion. They modulate several cellular mechanisms including proliferation, invasion, angiogenesis, polarity, and chemo resistance [5, 6]. Modification of integrin expression levels is contributed with both tumor progression and inhibition [7–9]. Up regulation of integrin subtypes such as β1, αv, and α4/5 are associated with tumor cell aggressiveness [10–13]. Integrin-associated intra-cellular signaling pathways are initially prompted by triggering focal adhesion kinase (FAK) to activate the Src kinases family that subsequently induce Ras/RAF/MEK/ERK or PI3K/Akt signaling pathways [14–16]. Integrin also induces cell proliferation through the GSK3β/Wnt axis [17]. Cisplatin (CDDP) as a widely used first-line chemotherapeutic drug accumulates in mitochondria and corrupts the mitochondrial function and structure [18], and therefore alters the level of metabolites associated with the glycolysis pathway and tricarboxylic acid cycle (TCA) cycle [19, 20]. Cisplatin can react with sulfhydryl groups of proteins and purine base in DNA. It binds with the purine bases to interfere with DNA repair that results in apoptosis induction in tumor cells [21, 22]. Cisplatin also promotes the generation of reactive oxygen species that induce apoptosis besides DNA damage [23]. Cisplatin is effective against the different tumor types including germ cell tumors, sarcomas, carcinomas, and lymphomas. However, there is a high rate of CDDP resistance and side effects in cancer patients [24]. Various cellular and molecular mechanisms such as drug efflux, detoxification, and increased DNA repair are involved in chemo resistance of tumor cells [25–29]. Integrins are involved in the drug resistance of various solid tumors [30–32] and hematological cancers [33–35] through modulation of the tumor cell interactions with interstitial matrix and ECM. Cell adhesion-mediated drug resistance (CAM-DR) facilitates prompt adaptation of tumor cells for extended survival [36]. ITGA7 inhibition reduced cisplatin resistance and sphere-forming ability of tumor cells [37]. MiR-199a-3p promoted the CDDP sensitivity of ovarian tumor cells through ITGB8 targeting [38]. ITGB1 induced tumor cell viability in various cancers by stimulating chemo resistance [30, 39, 40]. Several pre-clinical studies have already shown promising anti-tumor effects for integrin-associated treatments such as peptides, plant-derived molecules, and monoclonal antibodies. Moreover, the potential anti-tumor actions of a number of these agents have also been studied through clinical trials [41–44]. Monoclonal antibody against beta 1 integrin promoted the efficacy of cisplatin therapy in tumor cells [45]. Therefore, in the present review we discussed the molecular mechanisms of integrin protein family during CDDP response in tumor cells to introduce them as the reliable targets to improve the CDDP efficiency in cancer patients (Table 1). PubMed, Google Scholar, Scopus, and Web of Science were used for data retrieval that was limited to 15 Jan 2024. The following keywords were used: “integrin”, “cisplatin”, and “cancer”.

**Table 1:** Role of integrins in cisplatin response of tumor cells

Study	Integrin	Tumor Type	Samples	Integrin function	Clinical Application
lv [37]	ITGA7	Tongue	60 patients CAL-27, SCC-9, SCC-25, and HSC-4 cell lines	Increased CDDP resistance	Diagnostic/ prognostic
cui [38]	ITGB8	Ovarian	58 patients SKOV3 cell line	Increased CDDP resistance	Diagnostic/ prognostic
maiuthed [50]	ITGA4 ITGB1 ITGB5	Lung	H460 cell line	Increased CDDP resistance	Diagnostic
tian [55]	ITGB1	Hepatocellular	HepG2 and Bel-7402 cell lines	Increased CDDP resistance	Diagnostic
yin [56]	ITGB1	Breast	MDA-MB-231, MDA-MB-468, and HS578T cell lines	Increased CDDP resistance	Diagnostic
xu [57]	ITGB1	Esophageal	88 patients KYSE30, KYSE140, KYES150, KYSE180, KYSE40, KYSE450, KYSE510, TE10, and TE12 cell lines	Increased CDDP resistance	Diagnostic/ prognostic
yin [60]	αXβ2	Ovarian	SKOV3, HeyA8, OVCA429, OVCA433, and A2780 cell lines	Increased CDDP resistance	Diagnostic
wang [63]	ITGB5	Breast	MDA-MB-231 cell line	Reduce CDDP resistance	Diagnostic
xie [72]	ITGB1	Esophageal	278 patients KYSE150 and KYSE180 cell lines	Increased CDDP resistance	Diagnostic/ prognostic
zhu [75]	α5β1	Cervical	35 patients	Increased CDDP resistance	Diagnostic/ prognostic
li [81]	ITGA5	Laryngeal	94 patients LIU-LSC-1, AMC-HN-8, TU177, and LIU-LSC-1 cell lines	Increased CDDP resistance	Diagnostic/ prognostic
jia [87]	ITGB1	Lung	A549 cell line	Increased CDDP resistance	Diagnostic
chekenya [93]	α3β1	Glioblastoma	U251N, U87, and A172 cell lines	Increased CDDP resistance	Diagnostic
lin [101]	ITGB6	Ovarian	57 patients SKOV3 cell line	Increased CDDP resistance	Diagnostic/ prognostic
cao [103]	ITGB6	Bladder	155 patients 5637 and UC3 cell lines	Increased CDDP resistance	Diagnostic/ prognostic
sun [108]	αVβ6	Cholangiocarcinoma	18 patients TFK-1 and QBC939 cell lines	Increased CDDP resistance	Diagnostic/ prognostic
feng [117]	ITGB1	Oral	SCC-15 cell line	Increased CDDP resistance	Diagnostic
zhang [120]	ITGB3	Breast	87 patients MCF7, MDA-MB-231, and MDA-MB-435 cell lines	Increased CDDP resistance	Diagnostic/ prognostic
cheng [123]	ITGB3	Breast	87 patients HCC38, MDA-MB-231, and MDA-MB-468 cell lines	Increased CDDP resistance	Diagnostic/ prognostic
jang [126]	ITGB4 ITGA6	Oral	45 patients OC3, CGHNC9, and C9IV3 cell lines	Increased CDDP resistance	Diagnostic/ prognostic
hou [133]	ITGA5	Esophageal	ECa109 and TE-1 cell lines	Increased CDDP resistance	Diagnostic
chen [141]	α6β4	Breast	MDA-MB-231 cell line	Reduce CDDP resistance	Diagnostic
wu [146]	ITGB4	Colorectal	HCT116 and LOVO cell lines	Increased CDDP resistance	Diagnostic
andjilani [148]	ITGA6	Testicular	NCCIT cell line	Reduce CDDP resistance	Diagnostic
cataldo [149]	ITGA6	Breast	MDA-MB-231 and BT549 cell lines	Increased CDDP resistance	Diagnostic
rada [157]	α1β1	Ovarian	OVCAR3, CAOV3, ES2, and OV90 cell lines	Increased CDDP resistance	Diagnostic
irigoyen [159]	αVβ3	Lung	47 patients A549 and H1299 cell lines	Increased CDDP resistance	Diagnostic/ prognostic
baltes [160]	ITGB1	Breast	MCF7 and MDA-MB-231 cell lines	Increased CDDP resistance	Diagnostic

## PI3K/AKT signaling pathway

**Fig. 1:**
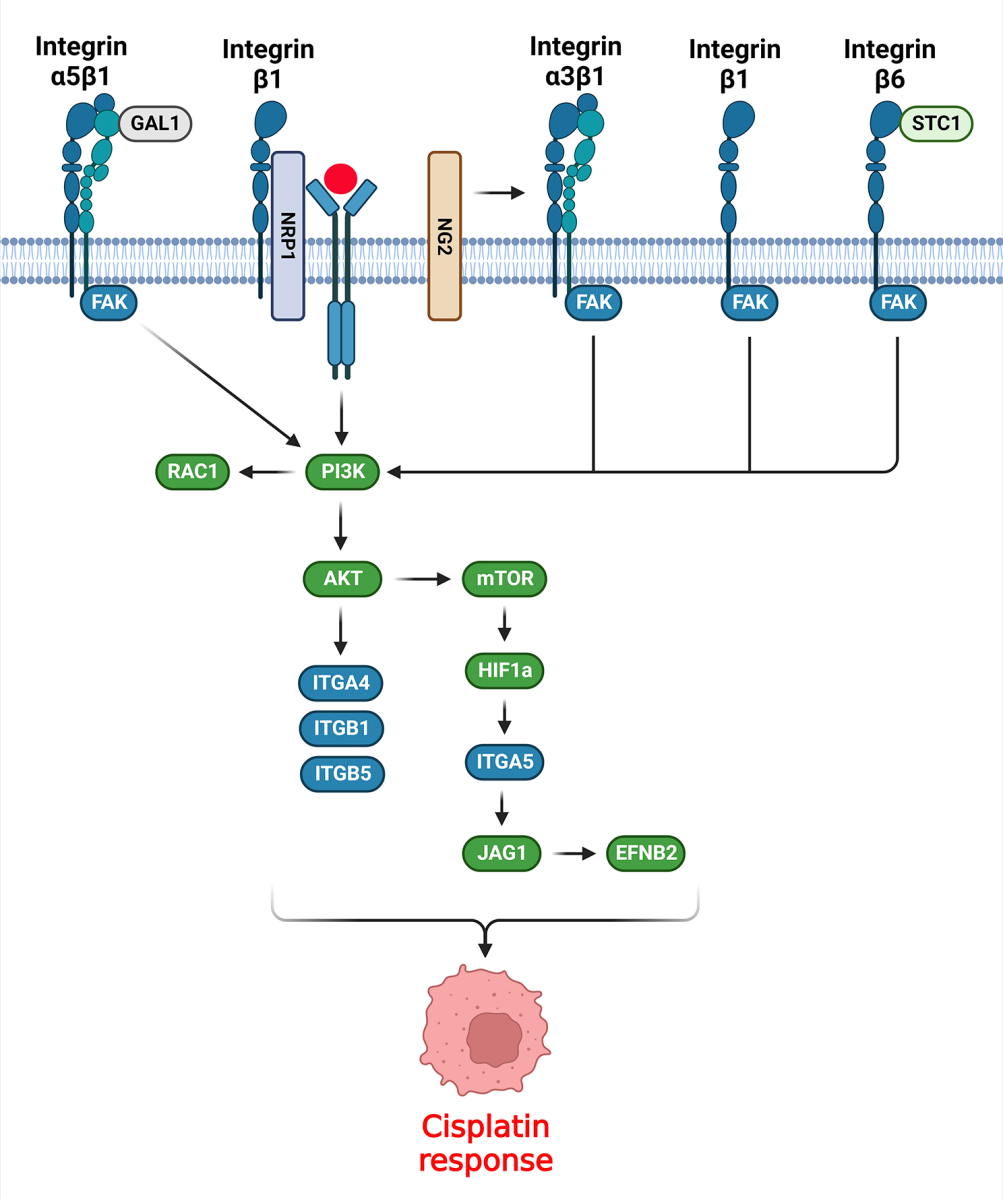
Role of integrins in cisplatin response by regulation of PI3K/AKT signaling pathway. (Created with *BioRender.com*)

Integrin protein family has a key role in CDDP response by regulation of PI3K/AKT pathway (Fig. 1). RhoA and Rac small GTPase are involved in regulation of actin rearrangements and cell migration [46–48]. RhoA-GTP which is the active from of RhoA enhances the aggregation of stress fibers and promotes new focal adhesions [49]. FAK activated AKT to induce actin polymerization and the formation of filopodia. Accordingly, CDDP promoted the FAK/AKT axis, which was associated with enhanced filopodia formation in cell lines. In addition, there was a dose-dependent over-expression of Rac-GTP and RhoA-GTP in CDDP-treated cells. Finally, CDDP-treated tumor cells have shown an enhanced activation of migratory proteins such as Rac, Rho A, AKT, and FAK and an over-expression of migration-associated integrins such as αv, α4, and β1/β5 [50]. Integrin β1 (ITGB1) interacts with focal adhesion kinase (FAK) to stimulate FAK phosphorylation [42, 51], subsequently triggering several signaling pathways such as integrin, c-Src, protein kinase B (Akt), and paxillin [52–54]. ITGB1 reversed the effects of 5-FU and CDDP in reducing proliferation and inducing apoptosis in HCC cells via FAK/Akt axis [55]. Integrin β1 up regulation was associated with higher stage, tumor recurrence, and poor prognosis in triple-negative breast cancer (TNBC) patients. Integrin β1 expression was also correlated with levels of phosphorylated AKT and FAK. Inhibition of Integrin β1 enhanced cisplatin sensitivity while reduced FAK and AKT activity in breast tumor cells [56]. Suppression of Integrin β1 inhibited esophageal squamous-cell carcinomas (ESCC) cell mobility as well as lymph nodal and pulmonary metastasis. Integrin β1 employed its pro-invasion role by modulating the FAK/Rac1 axis. In addition, ESCC cells lacking Integrin β1 were sensitized to CDDP treatment while remaining unresponsive to paclitaxel [57]. ECM1 modulates gastric tumor cell glucose metabolism and invasion via the ITB4/FAK/SOX2/HIF-1α axis [58]. Moreover, ECM1 modulates the architecture of actin cytoskeleton, which results in breast tumor cell invasion [59]. ECM1a modulated CDDP resistance and tumor formation by activating the AKT/FAK/Paxillin/Rac/cytoskeletal axis and stimulating the CD326 via various mechanisms such as alternative splicing through hnRNPLL, enhancing tumor cell stemness via ABCG1, and interacting with integrin αXβ2 through the Gly-Pro-Arg (GPR) motif. ECM1b stimulated myosin phosphorylation via inducing the AKT/FAK/Paxillin/Rac axis. Consequently, myosin phosphorylation is a potential participant in downstream of the ECM1/integrin αXβ2 axis. ABCG1 phosphorylated AKT2 to trigger the interaction of ECM1a and integrin αXβ2 [60]. ITGB5 stimulated intra-cellular pathways via promoting and utilizing integrin-related kinases such as FAK. FAK interacts with Src to participate in ITGB5-mediated response to Ras and VEGF transformation in fibroblasts [14, 61, 62]. Cisplatin inhibited cervical and breast tumor cell proliferation and growth by reducing the glucose metabolism. ITGB5 facilitated tumor cell glycolysis by promoting the FAK/p-FAK axis. ITGB5 significantly suppressed the anti-tumor properties of CDDP. ITGB5 promoted the FAK axis to stimulate tumor cell glycolysis while suppressed CDDP anti-tumor properties [63]. Integrin α5β1 is overexpressed in tumor vessel luminal surface and is involved in angiogenesis [64, 65]. Integrin α5β1 is contributed with tumor cell metastasis via modulating the matrix metalloproteinases (MMPs), cell adhesion, and actin cytoskeleton modification [66–69]. Integrin α5β1-induced cell adhesion triggers cell adhesion-mediated drug resistance (CAM-DR) and reduces the apoptosis and response of human myeloma cells to gamma-irradiation and DNA damage agents [70]. Proteins with Arg-Gly-Asp (RGD) binding region along with their integrin receptors have key roles in cellular adhesion. Integrin α5β1 is a main recognition receptor for RGD peptide motifs and Integrin β1 pathway is an important contributor to tension-associated metastasis [71]. There was Integrin α5β1 up regulation in ESCC tissues, which was correlated with poor prognosis. Inhibition of Integrin β1 and L1CAM diminished AKT activation and CDDP resistance [72]. Galectin-1 is a member of β-galactoside-binding lectins and contributes to many biological mechanisms including cell proliferation, cell invasion and adhesion, immunosuppression, and angiogenesis [73]. GAL1 induces tumor progression through activation of FAK/PI3K/AKT/mTOR axis [74]. There were integrin α5β1 and GAL1 up regulations in squamous cervical cancer stromal and tumor cells following neoadjuvant chemotherapy (NACT). In addition, patients with inadequate CDDP-based NACT response exhibited elevated levels of stromal integrin α5β1 and GAL1 as compared with controls. Therefore, integrin α5β1 plays a role in cervical cancer chemo resistance by inhibiting apoptosis [75].

mTOR is a serine/threonine kinase that is involved in autophagy, cell growth, ferroptosis, and metabolism by incorporating various components including nutrient status and growth factors [76–78]. EFNB2 is a ligand for Eph receptors that is involved in angiogenesis and tumor metastasis [79, 80]. ITGA5 stimulated tumor progression via up regulating EFNB2 in laryngeal squamous cell carcinoma (LSCC). There was mTORC1-ITGA5-EFNB2 axis up regulation in LSCC tissues, which was associated with poor prognosis. ITGA5 knockdown inhibited tumor growth while promoting CDDP sensitivity in LSCC cells. HIF-1α as an mTORC1 target was also found as a mediator of ITGA5 transcription in LSCC cells. Additionally, EFNB2 was found as an ITGA5 downstream gene in LSCC cells. ITGA5 knockdown in LSCC cells resulted in Jagged1 down regulation and Notch axis suppression. The mTORC1-mediated ITGA5 up regulation increased the levels of EFNB2 expressions via activation of the Jagged1/Notch axis in LSCC cells [81]. Neuropilin-1 (NRP1) acts as a co-receptor for VEGF during vascular formation and for class-3 semaphorin proteins through neuronal guidance [82–84]. Neuropilin-1 modulates neuronal cell migration and chemo repulsion by transducing semaphorin3A pathway via plexin-A1 [85, 86]. NRP1 antagonist suppressed the migration of ACHN and A549 carcinoma cells. NRP1 interacted with integrin-b1 to stimulate the adhesion of tumor cells to matrix proteins. NRP1 antagonist also enhanced the tumor cell sensitivity toward CDDP, paclitaxel, and 5-FU [87].

NG2 is a membrane associated proteoglycan produced by progenitor cells in various tissues [88]. NG2/MPG is implicated in multiple crucial mechanisms during the proliferation and metastasis of tumor cells such as cell responses to growth factors [89, 90] and cell migration [91, 92]. NG2/MPG induced chemo resistance by stimulating the PI3K/Akt axis through integrins. NG2/MPG-induced activation of α3β1 led to the transduction of survival signals by promoting the PI3K/Akt axis [93]. Adipocytes are abundantly found in the omentum to supply the fatty acids required for tumor growth. Therefore, targeting metabolism and transport of lipids might pave the way for new OC treatments [94]. STC1 is a glycoprotein hormone found in multiple tissues including the kidney [95], skeletal muscles [96], and spleen [97]. Elevated STC1 expression was seen in several cancers [98–100]. STC1 enhanced lipid metabolism by stimulating lipid-associated genes like perilipin1, TOM20, and UCP1. STC1 attached to ITGB6 to trigger the PI3K axis, which was mediated by FOXC2. Moreover, inhibition of STC1 and the FOXC2/ITGB6 pathway in ovarian cancer (OC) was associated with CDDP resistance [101]. Retinoic acid–related orphan receptor C (RORC) belongs to nuclear orphan receptors and acts as a DNA-binding factor [102]. There was RORC down regulation in bladder tumor tissues, which was associated with chemo-resistance and tumor stage. RORC was contributed with increased PD-L1 expression and abnormal signaling in bladder tumor cells. In addition, RORC modulated bladder tumor cell proliferation, chemo-resistance, and glucose metabolism by inhibiting the PD-L1/ITGB6/STAT3 pathway. RORC also enhanced CDDP sensitivity via the mitochondrial apoptosis axis [103].

Generally, integrins have a key role in CDDP resistance via direct activation of PI3K/AKT pathway by FAK/AKT axis. AKT also up regulates the integrins in a positive feedback. Moreover, integrins have indirect roles in CDDP resistance by up regulation of growth factors and subsequent increased activation of PI3K/AKT pathway.

## MAPK and Wnt/β-catenin signaling pathways

**Fig. 2:**
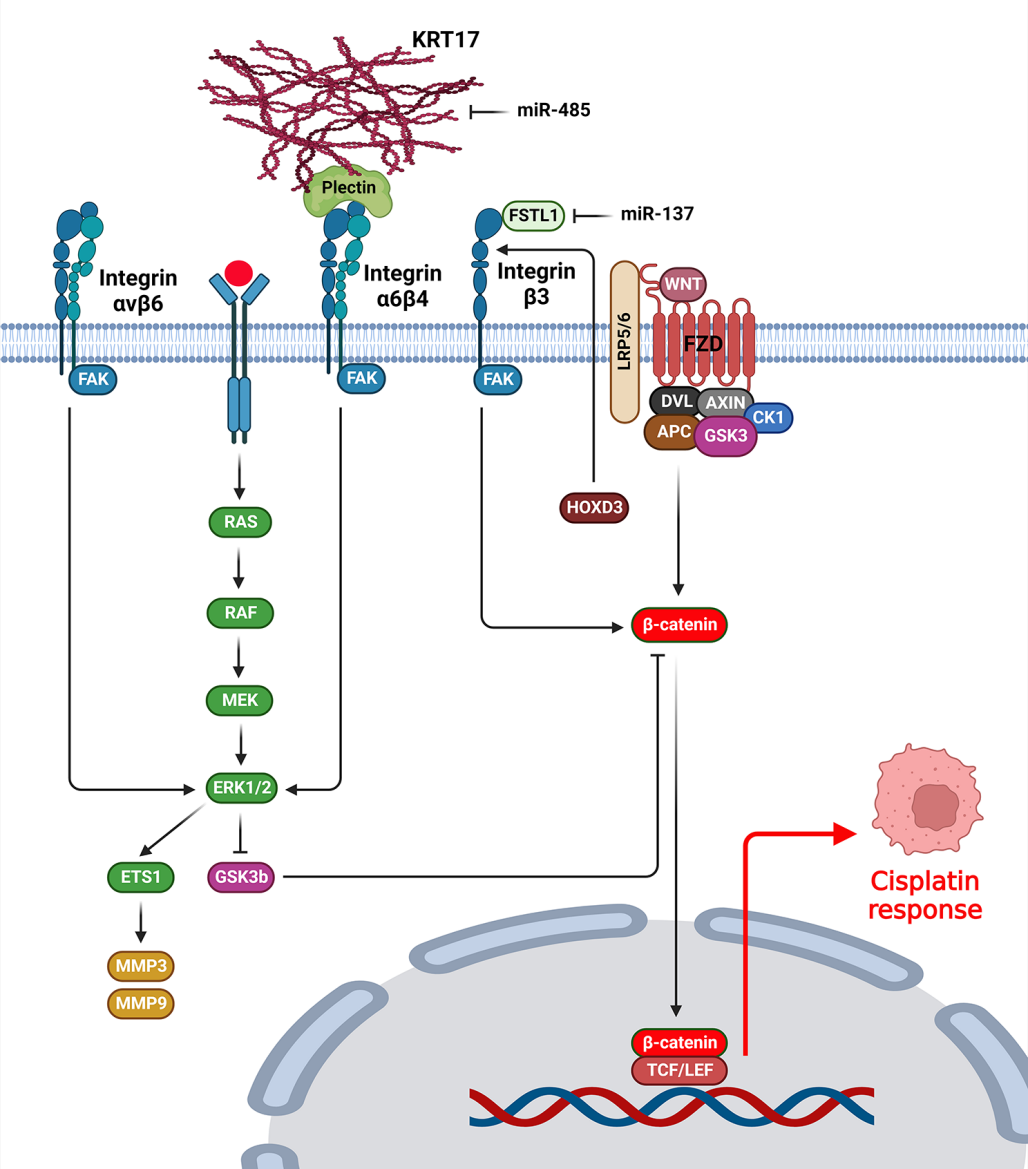
Role of integrins in cisplatin response by regulation of MAPK and WNT signaling pathways. (Created with *BioRender.com*)

Integrin protein family has a key role in CDDP response by regulation of MAPK and WNT signaling pathways (Fig. 2). Focal adhesion (FA) complex transfers the modulatory signals and mechanical forces between the ECM and cell [104]. Integrins, focal adhesion kinase (FAK), and Paxillin (PXN) constitute a pivotal part of FA complex. PXN triggers the activation of MAPK/ERK axis to promote CDDP resistance and Bcl-2 expression [105]. Integrin αvβ6 is exclusively expressed on normal epithelium, however it is also significantly over-expressed through tissue repair, morphogenesis, and tumor formation [106]. Integrin αvβ6 directly connects to ERK2 to prevent the cell phosphatase-induced deactivation of ERK2 that up regulates MMP-3 and MMP-9 by phosphorylating Ets-1 [107]. Integrin αvβ6 was associated with CDDP resistance, colony formation, and prognosis in hilar cholangiocarcinoma. Additionally, there was an integrin αvβ6 up regulation in hilar cholangiocarcinoma tissues, which was associated with advanced TNM stage, lymph nodal invasion, and poor differentiation. Moreover, integrin αvβ6 induced cisplatin resistance in cholangiocarcinoma cells via the ERK/MAPK axis [108]. Integrin αV subunit is found in numerous tumor cells and modulates cell-cell and cell-matrix interactions [109, 110]. It affects various cellular mechanisms such as apoptosis, survival, and proliferation [111–113]. Chloride intracellular channel 1 (CLIC1) acts as an ion protein channel that modulates apoptosis, cellular cycle, platelet release, neurogenesis, and bone formation [114, 115]. Chloride channels significantly participate in tumor development as it was reported that the involvement of some or potentially all chloride channels is crucial for tumor cell proliferation and metastasis [116]. Accordingly, CLIC1 was up regulated in oral squamous cell carcinoma (OSCC) tissues, which was correlated with tumor size, higher stage, and poor prognosis. CLIC1 knockdown mitigated OSCC cell proliferation and viability while enhancing apoptosis and CDDP response. CLIC1 inhibition diminished E-cadherin expression and incremented the levels of MMP-2, MMP-9, ITGB1, ITGαv, vimentin, and p-ERK. Moreover, an elevated expression of CASP3, CASP9, and p-p38 along with increased apoptosis were seen following CLIC1 inhibition. CLIC1 was contributed with OSCC cell metastasis by promoting MAPK/p38 and ERK signaling pathways through interacting with integrins [117].

Homeobox genes participate in oncogenesis by interacting with various signaling pathways [118, 119]. HOXD3 stimulated drug resistance and stemness in breast tumor cells via the Wnt/β-catenin axis through integrin β3 [120]. FSTL1 is a glycoprotein, which was initially recognized as a TGF-β1-inducible factor and a part of the family of Follistatin-SPARC [121]. However, FSTL1 interactions with signaling pathways extend beyond TGFβ. Multiple studies indicated that it participates in automodulatory feedback loops involving various factors such as IL1β, MMP-2, and BMPs [122]. MiR-137 was involved in the modulation of FSTL1/integrin β3/Wnt/β-catenin pathway. FSTL1 stimulated tumorigenesis by activating the Wnt/β-catenin axis via integrin β3. MiR-137 targeted FSTL1 to inhibit the Wnt/β-catenin that resulted in reduced drug resistance and breast tumor cell stemness [123]. Keratins are a subgroup of epithelial-specific intermediate filaments that modulate cellular adhesion and invasion by stabilizing hemidesmosomes via interacting with plectin/integrins complex [124, 125]. KRT17 promoted the integrin β4 expression. KRT17 regulated the integrin β4 and α6. KRT17 modulated OSCC drug resistance and stemness by regulating the integrin/FAK/Src/ERK/β-catenin axis. MiR-485-5p diminished sphere formation and reduced expression of CSC markers by KRT17 targeting [126]. ITGB4 was up regulated in secretory, classical, and primitive subgroups of lung squamous cell carcinoma (LUSC), which was associated with poor prognosis. ITGB4 targeting inhibited SOX2 expression in SOX2-expressing CSCs while enhancing cisplatin sensitivity. Accordingly, the knockdown of ITGB4 attenuated acetylation of H3K27 in the SOX2 promoter that resulted in SOX2 down regulation [127].

Generally, integrins activate the ERK that directly promotes CDDP resistance. ERK can also promote CDDP resistance by the inhibition of GSK3b that results in WNT signaling activation. Therefore, integrins can induce CDDP resistance by an interaction between the MAPK/ERK and WNT signaling pathways. On the other hand, integrins are also activators of the β-catenin that promotes CDDP resistance by WNT signaling pathway.

### DNA repair, apoptosis, and drug efflux

Homologous recombination (HR) and non-homologous end joining (NHEJ) are known as key repair pathways during response to cisplatin-mediated DNA damage [128]. HR acts as a high-fidelity repair axis that relies on a DNA template for the double-strand breaks (DSB) repair and therefore primarily functions during the G2 and S phases of the cellular cycle. In contrast, NHEJ functions without requiring a DNA template which allows it to act in any phase of the cell cycle but makes it more susceptible to errors [129]. BRCA1 as a key component of the HR pathway modulates cell proliferation and DNA damage repair by creating heterodimers with BARD1. Down regulation of BRCA1/BARD1 is correlated with the inhibition of DNA damage repair and enhanced sensitivity to DNA-damaging chemotherapy regimens in various tumors [99, 130, 131]. In addition, PI3K/AKT axis promotes DNA damage repair via up regulating BRCA1 and BARD1 [99, 132]. Integrin α5 regulated CDDP-induced apoptosis by up regulating BARD1 through the FAK/PI3K/AKT axis. Additionally, α5 inhibition sensitized the ESCC cells to CDDP by stimulating apoptosis and diminishing DNA damage repair [133]. Integrin α6β4 is known as a laminin receptor with a notably higher expression in TNBC compared with HER2-amplified or hormone-positive breast tumors [134]. Integrin α6β4 promotes p53 to cause Akt cleavage, p21 over-expression, and apoptosis [135–137]. 53BP1 is an important downstream effector of DNA-PK and p53 that can modify cisplatin sensitivity and is used for deciding between NHEJ and HR [138–140]. DNA-PK and p53 mutations are significantly involved in integrin α6β4-mediated CDDP sensitivity of TNBC cells. Integrin α6β4 promoted CDDP sensitivity while inhibited HR via modulating p53 and DNA-PK and accumulation of cells in the S phase. Additionally, integrin α6β4 was involved in CDDP sensitivity by stimulating 53BP1 phosphorylation through DNA-PK [141]. Direct interaction of two major elements of the FA complex including ITGB4 and PXN was associated with CDDP resistance in lung tumor cells. There was a cross-talk among molecules that modulate FA complex and EMT as it was shown that EMT-related pathways including TGF-β and Smad were associated with PXN and ITGB4 knockdown. ITGB4 and PXN were involved in the modulation of VDAC1 and USP1 transcriptions, which were contributed with CDDP sensitivity and tumor proliferation. Suppression of ITGB4/PXN or USP1/VDAC1 enhanced the oxygen consumption in mitochondria that resulted in elevated ROS formation and DNA damage. ITGB4/PXN suppression promoted CDDP sensitivity by inhibiting DNA repair processes via USP1 [142].

**Fig. 3:**
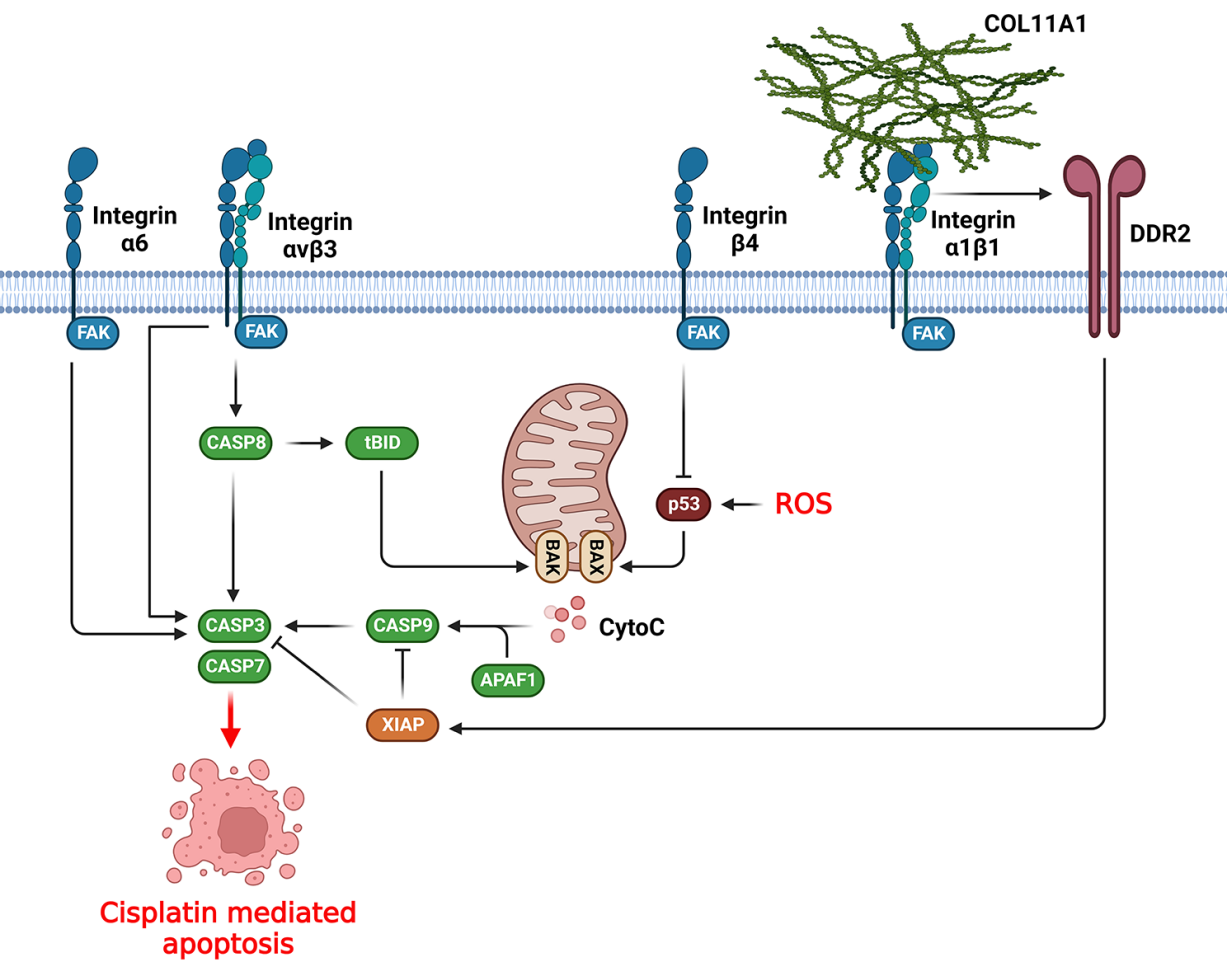
Role of integrins in cisplatin response by regulation of cisplatin mediated apoptosis. (Created with *BioRender.com*)

Integrin protein family has a key role in regulation of CDDP mediated apoptosis in tumor cells (Fig. 3). P53 activation is significantly involved in DNA repair, and suppression of p53 aggressive types results in DNA damage-mediated apoptosis [143–145]. Integrin β4 suppressed DNA damage-mediated activation of p53, while integrin β4 knockdown enhanced colorectal cancer (CRC) sensitivity to CDDP [146]. Throughout apoptosis-related signaling, CASP-8 and CASP-9 serve as initiator caspases and activate downstream genes such as CASP-3, CASP-6, and CASP-7 that degrade particular substrates including cytoskeletal DNA and proteins, and nuclear laminas to ultimately cause apoptosis [147]. α6-integrin promoted CDDP sensitivity and apoptosis in NCCIT cells via inducing adhesion of tumor cells to laminin and thereby activating apoptotic factors such as CASP-3, CASP-6, and AIF [148]. MiR-302b promoted CDDP response by regulating E2F/YY1/ITGA6 pathway in TNBC cells [149]. Collagens are major parts of the tumor microenvironment that interact with integrins to induce chemo resistance in several malignancies [30, 150–152]. Collagen type XI alpha 1 (COL11A1) is expressed mostly in recurrent and chemo-resistant ovarian cancers and also a subgroup of tumor-adjacent cancer associated fibroblasts (CAFs) [153–155]. Integrins and DDRs interact to increase integrin binding ability to collagens [156]. COL11A1 suppressed CDDP-induced apoptosis in ovarian tumor cells by promoting apoptosis inhibitor proteins such as BIRC2, BIRC3, and XIAP via discoidin domain receptor 2 (DDR2)-Src-PI3K/Akt-NF-kB axis through α1β1 integrin. COL11A1 induced IAP expression by activating NF-kB via the PI3K/Akt axis [157]. TGFβ1-induced protein (TGFBI) contributes to the binding of integrins to ECM components including fibronectin, collagen, and laminin, which is associated with the activation of cell adhesion, proliferation, and metastasis [158]. TGFBI up regulation was correlated with elevated chemo-sensitivity to several agents. Blocking the binding of TGFBI to αvβ3 integrin in Non-small cell lung cancer (NSCLC) cells reduced the TGFBI pro-apoptotic actions. TGFBI stimulated apoptosis in NSCLC cells via the proteolytic fragments derived from TGFBI. TGFBI-induced apoptosis occurred via the up regulation of caspase-3, caspase-7, and caspase-8 and the attachment of TGFBI-derived proteolytic fragments to αvβ3 integrin [159]. The α2β1-COL1 interaction down regulated ITGB1. There were BCRP, MRP1, and P-gp up regulations following tumor cell therapy with COL1. Additionally, a novel pathway was found in which ABC transporters were activated via ITGB1-mediated cell adhesion to COL1 in breast tumors [160]. PLOD2 acts as a crucial enzyme involved in collagen lysyl hydroxylation [161, 162]. PLOD2 enhances tumor metastasis by inducing Integrin β1 hydroxylation [163]. It also stimulates oral squamous cell carcinoma metastasis by activating Integrin β1 via regulating the IL-6/STAT3 axis [164]. PLOD2 knockdown repressed CD44 and CD133 levels and laryngeal cancer (LC) cell stemness. PLOD2 modulated tumorigenesis and LC cell stemness in cisplatin-resistant nude mice through Integrin β1. In addition, PLOD2 silencing reduced tumor progression and stemness in CDDP-resistant LC cells by suppressing P-gp and MRP1 through Integrin β1 [165].

Generally, integrins reduce CDDP-mediated apoptosis by activation of XIAP following the interaction with DDR2. They also inhibit the p53 to reduce Cytochrome C release from mitochondria that suppresses CDDP-mediated apoptosis in tumor cells. On the other hand, integrins can also promote the CDDP-mediated apoptosis by direct activation of CASP8 and CASP3. Therefore, integrins have dual functions in regulation of CDDP-mediated apoptosis that result in CDDP resistance in ovarian cancer while CDDP sensitivity in lung cancer.

## Conclusions

Integrins are involved in drug resistance of various solid tumors through modulation of the tumor cell interactions with interstitial matrix and ECM. Therefore, in the present review we discussed the role of integrin protein family in regulation of CDDP response in tumor cells. It has been shown that integrins mainly induced the CDDP resistance via interaction with PI3K/AKT, MAPK, and WNT signaling pathways. This review paves the way to suggest the integrins as the reliable therapeutic targets to improve CDDP response in tumor cells. However, more clinical trials and animal studies are required to use integrins as the therapeutic targets in cancer patients.

## Data Availability

The datasets used and/or analyzed during the current study are available from the corresponding author on reasonable request.

## Abbreviations

CAM-DR: Cell adhesion-mediated drug resistance
CLIC1: Chloride intracellular channel 1
CDDP: Cisplatin
CRC: Colorectal cancer
DDR2: Discoidin domain receptor 2
DSB: Double-strand breaks
ESCC: Esophageal squamous-cell carcinomas
ECM: Extracellular matrix
FAK: Focal adhesion kinase
FAK: Focal adhesion kinase
HR: Homologous recombination
ITGB1: Integrin β1
LSCC: Laryngeal squamous cell carcinoma
LUSC: Lung squamous cell carcinoma
MMPs: Matrix metalloproteinases
NACT: Neoadjuvant chemotherapy
NRP1: Neuropilin-1
NHEJ: Non-homologous end joining
OSCC: Oral squamous cell carcinoma
OC: Ovarian cancer
PXN: Paxillin
RORC: Retinoic acid–related orphan receptor C
TGFBI: TGFβ1-induced protein
TCA: Tricarboxylic acid cycle
TNBC: Triple-negative breast cancer
